# Female sex workers population size estimation in Rwanda using a three-source capture−recapture method

**DOI:** 10.1017/S0950268821000595

**Published:** 2021-03-18

**Authors:** G. Musengimana, E. Tuyishime, E. Remera, M. Dong, D. Sebuhoro, A. Mulindabigwi, E. Kayirangwa, S. S. Malamba, S. Gutreuter, D. Prybylski, R. H. Doshi, C. Kayitesi, V. Mutarabayire, S. Nsanzimana, P. Mugwaneza

**Affiliations:** 1 Rwanda Biomedical Center, Ministry of Health, Kigali, Rwanda; 2 Division of Global HIV/AIDS and TB, United States Centers for Disease Control and Prevention, Center for Global Health, Rwanda; 3 Division of Global HIV/AIDs and TB, United States Centers for Disease Control and Prevention, Center for Global Health, Atlanta, GA, USA; 4 United Nations Population Funds, UNFPA, Rwanda

**Keywords:** Capture−recapture, female sex workers, HIV/AIDS, population size estimation, sub-Saharan Africa

## Abstract

Establishing accurate population size estimates (PSE) is important for prioritising and planning provision of services. Multiple source capture−recapture sampling method increases PSE accuracy and reliability. In August 2018, the three-source capture−recapture (3S-CRC) method was employed with a stringent assumption of sample independence to estimate the number of female sex workers (FSW) in Rwanda. Using Rwanda 2017 FSW hotspots mapping data, street and venue-based FSW were sampled at the sector level of each province and tagged with two unique gifts. Each capture was completed within one week to minimise FSW migration between provinces and recall bias. The three captures had 1042, 1204 and 1488 FSW. There were 111 FSW recaptured between captures 1 and 2; 237 between captures 2 and 3; 203 between captures 1 and 3 and 46 captured in all three. The PSE for street and venue-based FSW in Rwanda lies within 95% credible set: 8328–22 806 with corresponding median of 13 716 FSW. The 3S-CRC technique was low-cost and relatively easy to use for PSE in hard-to-reach populations. This estimate provides the basis for determining the denominators to assess HIV programme performance towards FSW and epidemic control and warrants further PSE for home- and cyber-based FSW in Rwanda.

## Introduction

Globally, persons who exchange sex for money and other non-monetary items are at high risk of acquiring and transmitting HIV and other sexually transmitted infections. This risk is particularly high for those who exchange unprotected sex as their main source of income, such as female sex workers (FSW) [1]. FSW play a key role in HIV transmission to their male clients and subsequently, indirectly to the sexual partners of these male clients [2]. A systematic review conducted in 2012, reported the overall prevalence of HIV among sex workers in 26 low- and middle-income countries at 30.7%, with an odds ratio for HIV infection of 11.6 compared to other women of reproductive age [3]. The 2014 report from Joint United Nations Programme on HIV/AIDS (UNAIDS) stated that the prevalence of HIV among FSW was higher than the highest national value of HIV prevalence among the general population in nine countries including eSwatini, Botswana and Rwanda [4].

In Rwanda, the national HIV prevalence had reached a stable level of around 3% between 2005 and 2015 [5] for the population aged 15–49 years old. A recent national survey conducted in 2019 indicates a further decrease in national HIV prevalence (2.6%) [6] for Rwanda aged 15–49 years. However, HIV prevalence remains high among key populations, especially FSW [7]. The 2015 national bio-behavioural survey (BBS) reported that HIV prevalence has disproportionately affected FSWs (45.8%) in Rwanda, especially those from the capital city, Kigali (55.5%) [8]. It is essential to establish accurate population size estimates (PSE), in order to understand the magnitude and burden of HIV epidemic among FSWs to inform HIV prevention and treatment and guide resource allocation to this key population [9, 10].

However, sex work is structured differently around the world and comprehensively capturing and describing the characteristics of this heterogeneous population is making PSE difficult [10]. In most cases, sex work is stigmatised, or often illegal, which makes the FSW reluctant to disclose their source of income or profession [2]. In addition, criminalisation of sex work, discrimination and stigma keep FSW unidentifiable during daytime and some hold other occasional jobs. Additionally, their work is usually done during nighttime in nightclubs, hotels, bars and streets. Some proportion of FSW are home-based, which makes it even more difficult to include them in surveillance work or engaged them in HIV prevention and treatment services.

In the past decade, Rwanda conducted three PSE exercises among FSW using different methodologies: 2011, a household survey used the network scale-up method (NSUM) reporting the size of the FSW population ranged from 25 000 to 45 000 [11], and two hotspots mapping exercise in 2012 and 2017 reported 12 278 and 13 569 [12, 13]. In recent years, multiple source capture and recapture has been used for PSE, because it increases PSE accuracy with successive sampling, while other estimation methods face challenges to producing reliable estimates [14–17].

In 2018, Rwanda Biomedical Center (RBC) and US Centers for Disease Control and Prevention (CDC) conducted a PSE among FSW using three-source CRC (3S-CRC) methods to gain more accurate and reliable national estimates.

## Methods

### Capture recapture

In recent years, two-source CRC (2S-CRC) and 3S-CRC methods are also being used to estimate the sizes of hard to reach population such as FSWs, men who have sex with other men (MSM) and people who inject drugs (PWID) in many countries [14, 18–20].

Estimation is based on observations or ‘captures’ of population members on two occasions. The ‘captured’ population members are ‘marked’ by receiving a unique object during the first encounter. Population members who do and do not bear the mark are enumerated during the second encounter or ‘recapture’. The numbers of marks issued during the first encounter and the numbers of individuals who do and do not bear a mark are sufficient to estimate population size under the assumptions that: (1) the population members are neither added to or lost from the population during the sampling period; (2) the marks are identifiable with certainty and are not lost or duplicated and (3) each population member shares common fixed non-zero probability of encounter at both observation events and can be encountered independently with that probability. The first assumption of a fixed population size is unlikely to hold, but the adverse effects of violation are minimised by completing sampling over a short time interval. The second assumption is more vulnerable in human than in non-human populations because the latter are not able to refuse capture, and visible tags can be used as the marks. The third assumption is especially vulnerable in studies of human populations. Human behaviour is complex, and therefore it is difficult to assume that all humans will share a common, constant and non-zero encounter probability [18, 19].

The assumption that each population member shares a common, constant encounter probability can be relaxed, with three or more encounters, with estimation models including conventional log-linear models [21], and Bayesian non-parametric latent-class models [22].It is also possible to estimate three pair-wise 2S-CRC estimates from a 3S-CRC survey. We did this as a basis for comparison with 3S-CRC estimation. However, it is important to note that any resulting differences in estimates of population size are the combined result of the larger sample size from 3S-CRC and the relaxation of the untenable assumption required by 2S-CRC.

### Survey design

Data from the Rwanda 2017 hotspot mapping exercise for FSW size estimation conducted in 2017 in Rwanda^13^ were used as the sampling frame to generate a range of the number of objects to be distributed in each of 30 administrative districts in the five provinces of Rwanda.

A FSW was eligible if she met following criteria: (1) self-reported having sex with men in exchange for goods or money, (2) estimated age 15 years and above (based on visual observation of the key informants and objects distributors of this estimation) and (3) present at the visited venue/street at the time of data collection and identified by peer informants who worked in the same area. The age of consent in Rwanda is 18 years and above and because anyone under the age of 18 who is engaged in sex work is considered to be a victim of sexual exploitation, we included age 15–17 with a waiver of written informed consent from the local IRB. Including FSWs below 15 years would have required parental consent.

The objects used were small, inexpensive and branded with messages so they would have a memorable design and only be available from our staff who distributed them. The local civil society organisations were consulted to determine the type of objects and the unique messages in the local language (Kinyarwanda). A key holder with a message ‘Rinda Ubuzima’ (‘Protect your life’). and a bracelet with a message ‘♥ Ubuzima’ (‘Love your life’) were used for the initial two capture stages.

### Data collection

Thirty trained data collectors were assigned to the administrative sector within 30 districts in each province. In each sector, a data collector was paired with a local FSW key informant who served as a guide per sampling event. A total of 576 informants assisted as local guides. To achieve independence between captures, each data distributor and local guide were assigned to different sector hotspots in each capture stage. FSW who presented in the street and venue-based hotspots were approached by the data collectors and guides.

The 3S-CRC started with a capture stage (capture 1) where FSW encountered at hotspots were captured and tagged (marked) by accepting a unique object. After a one-week period, a second capture began during which FSW were asked if they had received unique objects during the previous week. If an FSW reported that she had received a unique object in the previous week, but could not show the object, the data collector showed her a laminated card with the distributed unique objects mixed with several incorrect objects. If the FSW correctly described and identified the object received in the last one week from the picture, she was considered as a recapture at capture 2. FSW who confirmed they have not received any objects or could not identify the object were considered newly captured.

The third capture was initiated one week after the second. Sampled FSW were approached and asked whether they had received unique gift(s) in the last two weeks. Visiting time depended on the days and hours selected by key informants for each venue/street. The estimation exercise was conducted in the first three weeks in August of 2018 in all five provinces.

### Data management and statistical analysis

We used 3S-CRC sampling to estimate the numbers of FSW in Rwanda using Bayesian non-parametric latent-class model [22], which eliminates the need to choose a single parametric log-linear estimation model. This simple extension of 2S-CRC requires only the distribution of a second mark during the second survey and the addition of a third survey during which population members are queried for receipt of the first mark during the first survey and the second mark during the second survey.

Data were collected with Android tablets that had a preprogramed Open Data Kit (ODK) form loaded (version 1.7). Data entered were sent in real-time to a server and backed up at midnight daily. Stata 15.0 and SAS 9.4 were used for sampling and descriptive analysis. We used the R 3.5.1 [23] RCapture 1.0 [24] package for exploration of heterogeneity in capture probabilities and for 2S-CRC estimation, and we used the LCMCR 0.3 [22] for 3S-CRC Bayesian non-parametric latent-class estimation. Three combinations of 2S-CRC estimates were calculated and were compared to the 3S-CRC estimates.

### Ethical consideration

This survey was reviewed and approved by the Rwanda National Ethics Committee. It was also reviewed in accordance with the US Centers for Disease Control and Prevention (CDC) human research protection procedures and was determined to be non-research. We received a waiver of informed consent for all participants because FSWs' age or other personal identifying information (PII) were not collected. Verbal consent was obtained as data collection was restricted to recording whether or not the FSWs accepted the gift/objects, at subsequent captures, whether FSWs can present/identify the gift they had received (age was estimated by the peer distributor). FSWs who were under 18 years (15–17) were provided with referral form to the nearest health facility for psychosocial support and HIV prevention and treatment intervention.

The encounter with a FSW took less than 5 min per person. The anonymity was ensured as no name of survey participants was collected on the distributor form and no other data tool was used. All data collected were retrieved from PDA and stored in a computer protected with a password. Employees exposed to privileged survey participant information, during implementation of the survey signed an Employee confidentiality agreement prior to the survey.

## Results

**Fig. 1.**
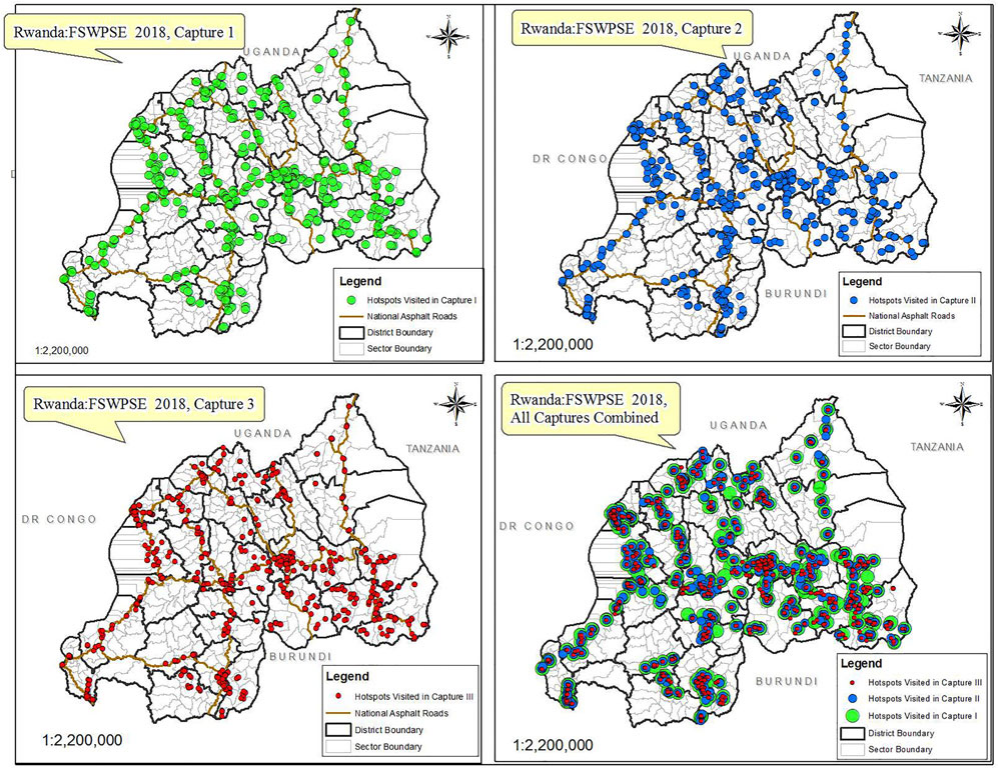
Maps indicating location of FSW hotspots that were visited during survey implementation in captures 1, 2 and 3, respectively.

A total number of 1042 unique objects were distributed countrywide during capture 1 and 1204 during capture 2. In a three-week survey implementation exercise, 192 sectors were visited countrywide in each capture. Figure 1 depicts maps showing venue/street hotspots visited in each of the three capture stages (Fig. 1).

### Descriptive analysis

Among 1135 women approached during capture 1, 1080 (95.2%) self-reported to be FSW, 1071 (99.2%) of them were offered the object and 1042 (97.3%) accepted it. In capture 2, 1278 women were approached, among them 1234 (96.6%) self-reported to be FSWs, 7 (0.6%) of them reported having been approached in the same capture with the same object (defined as those who were captured in a different hotspot in the same capture), 1227 (99.4%) were approached for the first time in capture 2. Among those approached in capture 2, objects acceptance rate was higher 1204 (98.1%) compared to capture 1. During capture 3, 1515 women were approached, 1494 (98.6%) self-reported to be FSW and among them 1488 (99.6%) answered questions about having received the object in previous captures and counted as captured (Table 1).

**Table 1.** Description of approached FSWs during captures 1, 2 and 3 with corresponding percentages

	Capture 1	Capture 2	Capture 3
	*n* (%)	*n* (%)	*n* (%)
FSWs approached	1135	1278	1515
Self-reported to be FSWs			
Yes	1080 (95.2)	1234 (96.6)	1494 (98.6)
No	55 (4.8)	44 (3.4)	21 (1.4)
Already in current capture			
Yes	9 (0.8)	7 (0.6)	6 (0.4)
No	1071 (99.2)	1227 (99.4)	1488 (99.6)
Unique object acceptance			
Accepted	1042 (97.3)	1204 (98.1)	N/A
Refused	29 (2.7)	23 (1.9)	N/A
**FSWs presumed to be under 18 years**	**33**	**12**	**15**
Accepted referral form	4 (12.1)	10 (83.3)	9 (60.0)
Refused referral form	29 (87.9)	2 (16.7)	6 (40.0)

**Fig. 2.**
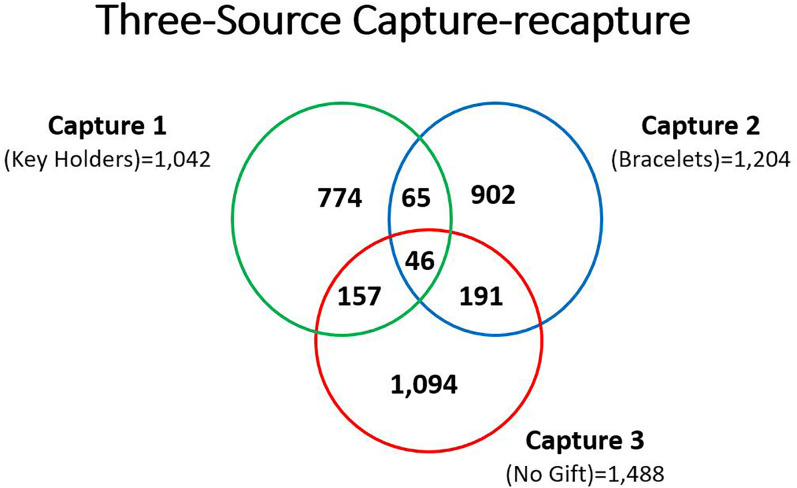
Venn diagram indicating the overlaps in all captures, total number of captures and recaptures for FSWs in Rwanda, 2018.

The results for the numbers of single, double and triple captures are presented in Venn diagram in Figure 2. These numbers were used by Bayesian 3S-CRC approach, the non-parametric latent class models, to estimate the population size of FSW in Rwanda. The overlaps were determined by counting the number of FSW common to both captures or to all three captures (Fig. 2).

### FSWs population size estimation using a 2s-CRC

The PSE and uncertainty intervals with their corresponding 95% confidence intervals (CI) were calculated using the exact hypergeometric method presented in Table 2. The FSWs population estimates using capture 1 and two were 9167, 95% CI (7675–10659) and capture 1 and three were 5893, 95% CI (5241–6545). The estimates increased with adjusted estimates considering the 576 FSWs that were used as study guides (key informants), (Fig. 2).

**Table 2.** FSWs population size using 2S-CRC method

	Captures	FSW population size (95% CI)	Adjusted median ( + 576 FSW guides) (credible set)	Method/Models
*2S-CRC*	1 and 2:	9167 (7675–10 659)	9743 (8251–11 235)	Exact hypergeometric method
	1 and 3:	5893 (5241–6545)	6469 (5817–7121)	
	2 and 3:	5926 (5327–6525)	6502 (5903–7101)	

CI, Confidence intervals.

### FSWs population size using 3S-CRC method

**Fig. 3.**
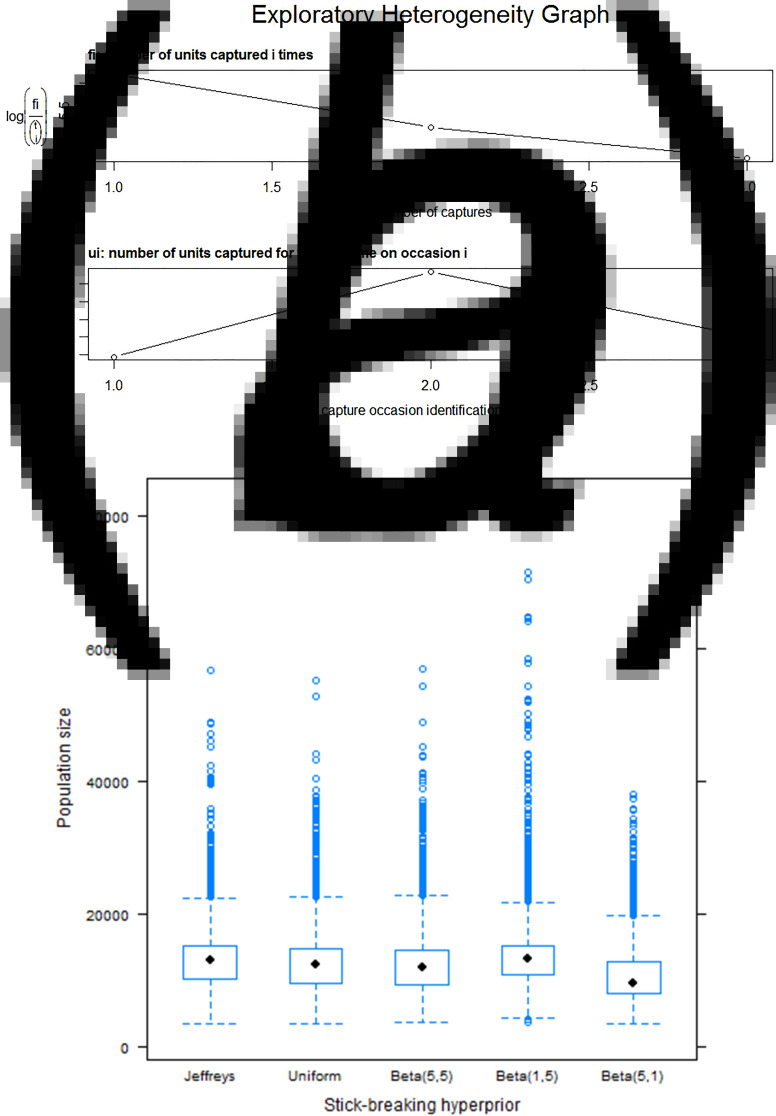
(a) Exploratory heterogeneity graph, (b) sensitivity analysis for prior probability distribution selection.

To check for heterogeneity assumption, an exploratory heterogeneity graph was plotted. In Figure 3, the ***f***_***i***_ plot is convex-up, suggesting individual heterogeneity in capture probabilities (25); the ***u***_***i***_ plot in the same Figure 3a, is distinctly non-linear, suggesting additional heterogeneity (Fig. 3a). After testing for heterogeneity assumption of selection probability across the three captures and found that they were heterogeneous (Fig. 3a), Bayesian latent class model was fitted. We evaluated the sensitivity of the estimates of population size to hyperprior choices given by the beta (1, 1) (uniform), beta (0.5, 0.5) (Jeffrey's prior), beta (5, 5), beta (1, 5) and beta (5, 1) distributions the stick-breaking process which contains the number of latent classes. The latter three are strongly informative. We chose the Jeffrey's prior as it is non-informative, therefore resulting in estimates that are totally reliant on the data (Fig. 3b).

After fitting the Bayesian model, the population size of street and venue-based FSWs in Rwanda was estimated to be within a credible set ranging from 7752 to 22 230 with corresponding median of 13 140 (Table 3). As the number of FSW guides (576) that facilitated the data collection is known and fixed, hence this number was added onto the estimates generated by the Bayesian model (Table 3).

**Table 3.** Three sources capture−recapture estimates (adjusted and unadjusted) from the Bayesian model

	Unadjusted estimates	
	Median	95% Credible set
3S-CRC	13 140	7752–22 230
	Estimates adjusting for 576 FSW guides that facilitated the data collection	
	Median	95% Credible set
3S-CRC	13 716	8328–22 806

## Discussion

The Rwanda FSW PSE 2018 data provide a national estimate of the FSW population. This nationwide FSW PSE using a 3S-CRC method estimated a credible set ranging from 8328 to 22 806 with a median of 13 716 FSW. This is the first time a 3S-CRC method was used to estimate the population size of FSW at the national level in Rwanda. As of January 2019, HIV prevention program data indicate that, approximately 15 000 FSW were enrolled in a key population program in 18 districts of Rwanda. In this study, a population size of FSW was estimated to range from 8328 to 22 806 FSW. Given the number of FSW enrolled in HIV prevention programme, using the upper bound of the credible set provides the best-case scenario for FSW programming.

In 2010, Rwanda's FSW PSE was estimated using multiple methods namely capture−recapture, enumeration and multiplier methods, and generated estimates of 3205, 3348 and 2253 FSW, respectively [25]. In 2011, a PSE of FSW was conducted through a household survey which estimated the FSW population between 25 000 and 45 000 using the network scale up method [11]. In 2012, a participatory site assessment method adapted from the mapping and census method for FSW PSE resulted in 12 278 FSW being approximated. In 2017, the RBC conducted a new round of national FSW hotspot mapping exercises with a result of 13 569 for estimated size for home-, street- and venue-based FSW [12, 13].

Although these previous four PSE for Rwanda FSW conducted since 2010 used different methods and cannot be directly compared, they can be triangulated with other estimates to better estimate the population size of FSW in Rwanda. In addition, size estimation conducted in Côte d'Ivoire, Western Kenya, Nigeria and Iran used 2S-CRC [15–17], while our survey included one additional capture 3, which is comparable to the study from Uganda [14]. This 2018 estimate derived from a 3S-CRC method using Bayesian latent class approach, has advantage over the others because it produces credible sets (Bayesian probability intervals), which better reflect the true level of model selection uncertainty and do not depend on large samples for validity. Credible sets are naturally based on the posterior distribution of the population size given the data and priors. The Bayesian latent-class approach incorporates model uncertainty in a natural and mathematically defensible way.

One of critical assumption for CRC experiments is that the population is closed during capture period. However, FSW are dynamic – both in time/space, and membership, they can move from one province to the other, change hotspots often or leave FS work at anytime. To minimise the effects, the estimation exercise was implemented within a short period where all the three captures were completed within three subsequent weeks. Because current estimates are limited to FSW who congregated at hotspots (i.e. street and venue-based FSW), the FSW who do not congregate at hotspot venues, such as home-based FSW, were not able to be included. A survey, such as an immediate follow-up BBS survey would provide the visibility of non-home-based FSW and improve the estimating results.

We anticipate underreporting of FSW because this profession is illegal in Rwanda and subject to a certain level of stigma and discrimination. In addition, the study was designed to generate national level estimates and cannot support reliable subnational estimation. Furthermore, FSWs were not interviewed or asked questions during data collection, this might have caused some error.

Size estimation of key populations at risk of HIV (e.g. FSW and their clients) is essential for understanding the magnitude and burden of the HIV epidemic and estimating the gap in the coverage of HIV prevention programme in these populations. Knowing the estimated size of FSW in Rwanda enables HIV prevention programmes targeting FSW to estimate the proportion of FSW living with HIV aware of their HIV status. This will inform the development of appropriate prevention and treatment programmes, measurement of service coverage and informing strategic planning and resource allocation to reach FSWs with HIV services.

## Conclusions

We estimate that the population size of FSW in Rwanda ranges from 8328 to 22 806. This estimate provides a basis for determining the denominators to assess HIV program performance among FSW in Rwanda relative to UNAIDS 90-90-90 targets. This estimate also provides the size of FSWs to be targeted for HIV prevention programmes because we know how many we need to support. The current estimate underestimates the total number of FSW because it is limited to street and venue-based FSW. In the future, an improved FSW-PSE should be conducted to overcome this limitation and gain more precise estimates for Rwanda.

## Data Availability

The data that support the findings of this study are available on request from the Rwanda Ministry of Health though Rwanda Biomedical Centre using the email: eric.remera@rbc.gov.rw.
